# Relationship Between Linezolid Exposure and the Typical Clinical Laboratory Safety and Bacterial Clearance in Chinese Pediatric Patients

**DOI:** 10.3389/fphar.2022.926711

**Published:** 2022-08-01

**Authors:** Ben-Nian Huo, Yue-E. Wu, Ling Shu, Ruo-Qi Zhang, Jian-Wen Xiao, Qian-Bo Li, Wei Zhao, Yun-Tao Jia, Lin Song

**Affiliations:** ^1^ Ministry of Education Key Laboratory of Child Development and Disorders, Chongqing Key Laboratory of Pediatrics, Department of Pharmacy, National Clinical Research Center for Child Health and Disorders, Children’s Hospital of Chongqing Medical University, Chongqing, China; ^2^ Key Laboratory of Chemical Biology (Ministry of Education), Department of Clinical Pharmacy, School of Pharmaceutical Sciences, Cheeloo College of Medicine, Shandong University, Jinan, China; ^3^ State Key Laboratory of Southwestern Chinese Medicine Resources, Key Laboratory of Standardization for Chinese Herbal Medicine, School of Pharmacy, Ministry of Education, Chengdu University of Traditional Chinese Medicine, Chengdu, China; ^4^ Department of Hematology, Children’s Hospital of Chongqing Medical University, Chongqing, China; ^5^ Department of Information Center, Children’s Hospital of Chongqing Medical University, Chongqing, China; ^6^ NMPA Key Laboratory for Clinical Research and Evaluation of Innovative Drug, Qilu Hospital of Shandong University, Shandong University, Jinan, China

**Keywords:** linezolid, exposure, safety, efficacy, paediatric

## Abstract

**Objectives:** There have been limited studies concerning the safety and efficacy of linezolid (LZD) in children. This study aimed to evaluate the association between LZD exposure and clinical safety and efficacy in Chinese pediatric patients.

**Methods:** This retrospective cross-sectional study included patients ≤18 years of age who received ≥3 days of LZD treatment between 31 January 2015, and 31 December 2020. Demographic characteristics, medication information, laboratory test information, and bacterial culture results were collected from the Hospital Information System (HIS). Exposure was defined as AUC_24_ and calculated by the non-linear mixed-effects modeling program (NONMEM), version 7.2, based on two validated population pharmacokinetic models. Binary logistic regression analyses were performed to analyze the associations between AUC_24_ and laboratory adverse events, and receiver operating characteristic curves were used to calculate the cut-off values. Efficacy was evaluated by bacterial clearance.

**Results:** A total of 413 paediatric patients were included, with an LZD median (interquartile range) dose, duration, clearance and AUC_24_ of 30.0 (28.1-31.6) mg/kg/day, 8 (4‒15) days,1.31 (1.29-1.32) L/h and 81.1 (60.6-108.7) mg/L·h, respectively. Adverse events associated with TBil, AST, ALT, PLT, hemoglobin, WBC, and neutrophil count increased during and after LZD treatment when compared with before medication (*p* < 0.05), and the most common adverse events were thrombocytopaenia (71/399, 17.8%) and low hemoglobin (61/401, 15.2%) during the LZD treatment. Patients with AUC_24_ higher than 120.69 mg/L h might be associated with low hemoglobin 1–7 days after the end of the LZD treatment, and those with an AUC_24_ higher than 92.88 mg/L∙h might be associated with thrombocytopaenia 8–15 days after the end of the LZD treatment. A total of 136 patients underwent bacterial culture both before and after LZD treatment, and the infection was cleared in 92.6% (126/136) of the patients, of whom 69.8% (88/126) had AUC_24_/MIC values greater than 80.

**Conclusion:** Hematological indicators should be carefully monitored during LZD treatment, especially thrombocytopaenia and low hemoglobin, and a continuous period of monitoring after LZD withdrawal is also necessary. Since the AUC_24_ cut-off values for laboratory adverse events were relatively low, a trade-off is necessary between the level of drug exposure required for treatment and safety, and the exposure target (AUC_24_/MIC) in pediatric patients should be further studied, especially for patients with complications and concomitant medications.

## Introduction

LZD is an oxazolidinone antibiotic that inhibits bacterial protein synthesis and prevents bacterial reproduction by binding to the bacterial 23S ribosomal RNA of the 50S subunit blocking the formation of a functional 70S initiation complex (Daniel and Ronald, 2001). The absolute oral bioavailability of LZD is approximately 100%, with good tissue penetration and non-susceptibility to drug resistance (Roger et al., 2018). It is commonly used to treat severe Gram-positive bacterial infections. It is considered clinically effective but is usually difficult to manage because of its large individual pharmacokinetic differences and related adverse events, especially in pediatric patients, and it is an antibiotic with a narrow therapeutic window and dose-dependent toxicity (Sotgiu et al., 2012; Peyrani et al., 2014).

A meta-analysis showed that approximately one out of every two patients experienced adverse events due to LZD (4), but the incidence of LZD-related adverse reactions in Chinese children has rarely been reported. Hematological toxicity, hyperlacticaemia, and optic neuropathy are the main adverse reactions to LZD (3, 4, 5), and thrombocytopaenia is a significant adverse drug reaction with the highest risk in the clinic (Han et al., 2021). The incidence of LZD-induced thrombocytopaenia varies from 3.8% to 15.7% in children worldwide (Meissner et al., 2003; Garazzino et al., 2011; Garazzino and Tovo, 2011), which is lower than that in adults (range 16.7–60.5%) but higher than the drug label reported (2.4% in children) (Natsumoto et al., 2014; Hirano et al., 2014; U.S. Department of Health and Human Services, 2021). In addition, the risk of adverse reactions increased with exposure and duration of LZD treatment (Matsumoto et al., 2014; Rao et al., 2020), the incidence of thrombocytopaenia in adult patients was significantly higher when the trough concentration was greater than 7.5 mg/L (Nukui et al., 2013), and children with thrombocytopaenia had a significantly higher average trough concentration than those without thrombocytopaenia (19.8 vs. 6.8 mg/L) (Ogami et al., 2019), but the relationship between LZD exposure and adverse reactions in Chinese children has not been studied.

Population pharmacokinetic models of LZD in children have been widely established and have been used to calculate drug exposure, and their extrapolated predictive performance has been confirmed (Vinks, 2002; Jungbluth et al., 2003; Rao et al., 2020; Ogami et al., 2021). Therefore, in this study, we aimed to calculate LZD exposure using population pharmacokinetic models of LZD in children and then evaluated the relationship between drug exposure and adverse events in Chinese pediatric patients. The efficacy of LZD was also evaluated for personalized drug therapy using LZD and risk assessment in clinical therapy.

## Patients and Methods

### Study Design

We conducted a retrospective cross-sectional study of hospitalized children who received LZD treatment in the Children’s Hospital of Chongqing Medical University (Chongqing, China) from 31 January 2015, to 31 December 2020. This study was approved by the Ethics Committee of the Children’s Hospital of Chongqing Medical University with an informed consent exemption considering the observational and retrospective nature of the study, and the data were collected without identifiers (Approval No. 2020–282). We used the STROBE checklist as the main reference in reports of this cross-sectional study.

### Study Subjects

Patients younger than 18 years of age that were intravenously or orally administered LZD for at least three consecutive days were included. The criteria for patient exclusion were a lack of demographic data, LZD medication information, or baseline laboratory data for safety assessment.

### Data Collection

Medical records in the hospital information system (HIS) database of the patients who matched the inclusion criteria were extracted by an information centre engineer, and then, two of the authors manually screened the information for inclusion and identified and recorded reasons for exclusion. Any disagreements were resolved through discussion or by consulting a third author. The HIS database is a comprehensive, integrated information system includes detailed clinical and demographic information about all pediatric patients, and the information from the HIS database are derived from daily notes recorded by clinicians of all the patients, which helps to improve patient care by assessing data and making recommendations for care. The following information of the included patients was extracted and recorded.

1) Demographic parameters, medication information, and serum creatinine concentration (Scr): sex, age, body weight, height, clinical diagnosis, LZD medication route, dosage, administration time and duration of LZD treatment, and serum creatinine concentrations measured during LZD medication. These data were used for the LZD exposure calculations.
2) Data for safety assessment: total bilirubin (TBil), aspartate aminotransferase (AST), alanine aminotransferase (ALT), Scr, blood urea nitrogen (BUN), platelet (PLT), hemoglobin, white blood cell count (WBC), and neutrophil count measured before LZD medication, during treatment, and 1–7 days and 8–15 days after the last dose of LZD administration.
3) Data for efficacy assessment: bacterial culture results and the measured LZD minimal inhibitory concentration (MIC) values.

### Exposure Analysis

Two population pharmacokinetic models established by Garcia-Prats AJ et al. (Garcia-Prats et al., 2019) and Si-Chan Li et al. (Li et al., 2019) were chosen to calculate the exposure of LZD in this study, and the predictive performance of the two models has been validated and used in our hospitals. The NONMEM, version 7.2 (Icon Development Solutions, Columbia, MD, United States), was used to perform the simulations and calculate the LZD exposure. The related formulas of the two models are shown in Supplementary Table S1. Exposure to LZD was defined as a 24-h area under the concentration-time curve (AUC_24_) in the steady state, and AUC_24_ = daily dose/clearance.

### Safety and Efficacy of LZD

In this study, the safety of LZD was evaluated by laboratory adverse events, which were defined based on the Food and Drug Administration label for LZD (13), see Table 1. Efficacy was evaluated by comparing the bacterial culture results before and after LZD treatment, and the proportion of AUC_24_/MIC values greater than 80 was also calculated, as previous studies have shown that higher success rates for LZD might occur at AUC_24_/MIC values greater than 80 (22,23,24,25).

**TABLE 1:** The definition of laboratory adverse events^a^.

	Normal Baseline Values^b^	Abnormal Baseline Values
Liver dysfunction		
High TBil	>2 times of ULN	>1.5 times of the baseline value
High AST	>2 times of ULN	>2 times of the baseline value
High ALT	>2 times of ULN	>2 times of the baseline value
Renal dysfunction		
High Scr	>2 times of ULN	>2 times of the baseline value
High BUN	>2 times of ULN	>2 times of the baseline value
Hematology properties		
Thrombocytopenia	<75% of LLN	<75% of LLN
Low hemoglobin	<75% of LLN	<75% of LLN and <90% of baseline value
Low WBC	<75% of LLN	<75% of LLN
Low neutrophils	<50% of LLN	<50% of LLN

a Laboratory adverse events were defined based on the Food and Drug Administration label of linezolid, and the corresponding reference is 13.

b LLN, and ULN, values of each parameter were considered based on the normal baseline value ranges defined by the department of clinical laboratory in our hospital.

Abbreviations: ALT, alanine amimotransferase; AST, aspartate aminotransferase; BUN, blood urea nitrogen; LLN, lower limit of the normal; Scr, serum creatinine concentration; TBil, total bilirubin; ULN, upper limit of normal; WBC, white blood cell count.

### Statistical Analysis

The Shapiro–Wilk test was performed to assess whether the data were normally distributed. Continuous outcomes with abnormal distributions are expressed as medians and interquartile ranges. Categorical outcomes are reported as counts and percentages. AUC_24_ was calculated to indicate the *in vivo* exposure of LZD, and patients were divided into four groups according to the interquartile range (IQR) of AUC_24_: quartile 1, quartile 2, quartile 3, and quartile 4. A generalized estimating equation (GEE) was used to analyze the incidence of changes in the laboratory adverse events over time before and after LZD treatment. Binary logistic regression analyses were performed to analyze the associations between AUC_24_ and safety, and age, sex, and laboratory parameters measured before the LZD medication were considered potential confounding factors based on the preliminary analysis and a literature review (Chang et al., 2013; Mullins et al., 2013; Chuang et al., 2014) and were included for adjustment. The covariates were evaluated continuously, and by the quartile of exposure, odds ratios (ORs) and 95% confidence intervals (CIs) were calculated. Receiver operating characteristic (ROC) curves were used to estimate the exposure cut-off values for the laboratory adverse events. The sensitivity and specificity and the maximum Youden’s index of the ROC curve were calculated, and the maximum Youden’s index was selected as the optimal exposure cut-off value. Youden’s index equals the result of subtracting one from the sum of sensitivity and specificity (Rui et al., 2016). *p* values less than 0.05 were considered to be statistically significant. Data were gathered using the Microsoft Excel software (Redmond, WA, United States), and all analyses were performed using the IBM SPSS statistical software package, version 22.0 (SPSS, Chicago, IL, United States).

## Results

### Patient Characteristics

**FIGURE 1:**
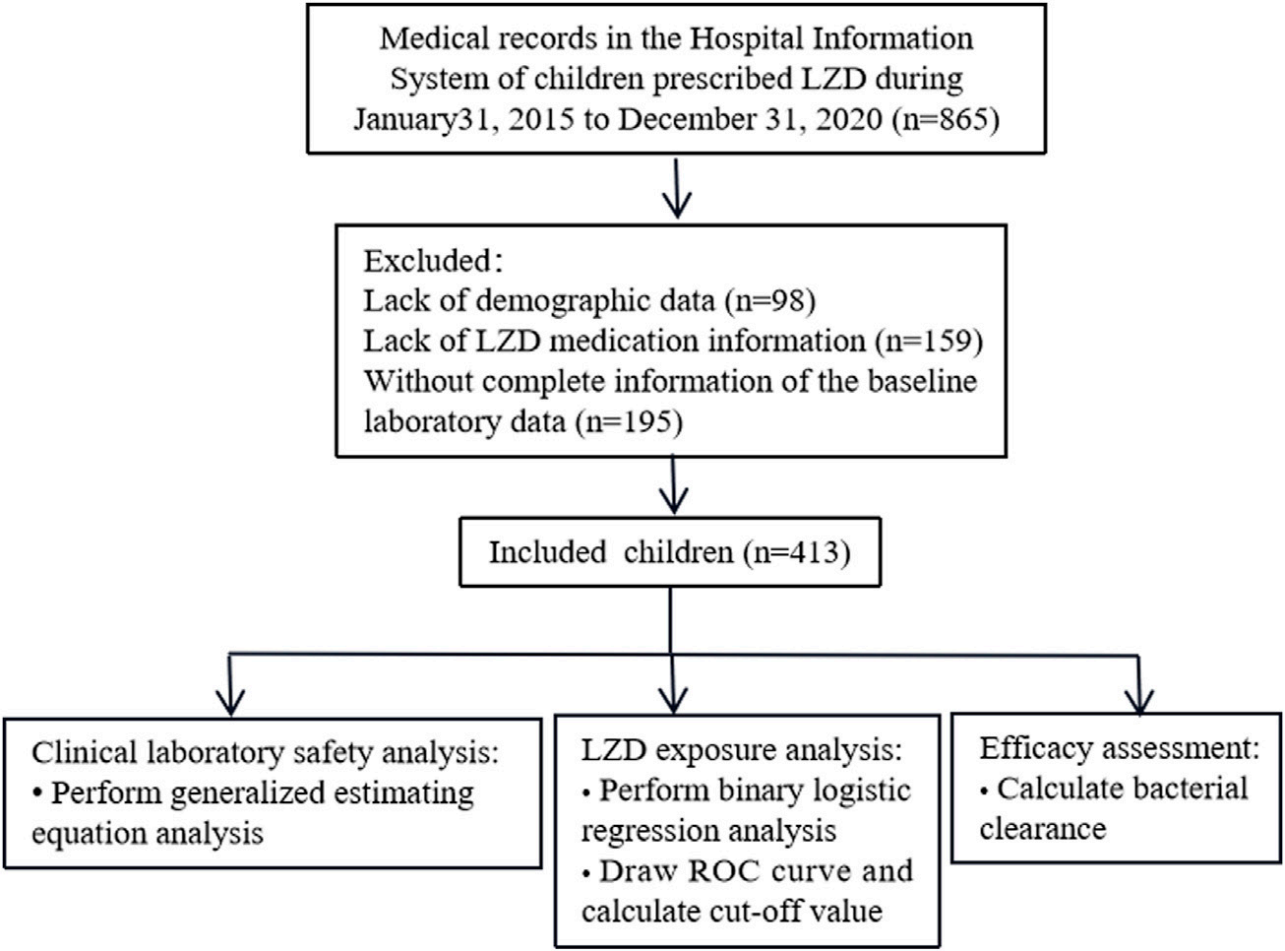
Flow chart for patient inclusion.

Medical records of 865 patients who received LZD therapy were extracted and screened, and 413 patients who met our inclusion criteria were included in this study (Figure 1). Demographic characteristics, medication information, and exposure to LZD after drug administration are shown in Table 2. Counts and percentages of the laboratory adverse events are shown in Table 3. The most common adverse events were thrombocytopaenia (71/399, 17.8%) and low hemoglobin (61/401, 15.2%) during LZD treatment.

**TABLE 2:** Demographic characteristics, medication information, and exposure of linezolid according to the AUC_24_ quartile (*n* = 413).

	Total	Quartile 1	Quartile 2	Quartile 3	Quartile 4
Number of participants	413	106	102	102	103
Characteristic					
Age, year, median (IQR)	1.2 (0.4–3.6)	0.2 (0.1–0.4)	0.9 (0.5–1.2)	2.0 (1.1–2.9)	6.0 (4.0–9.7)
Sex, n (%)					
Men	252 (61.0)	53 (50.0)	64 (62.7)	67 (65.7)	68 (66.7)
Women	161 (39.0)	53 (50.0)	38 (37.3)	35 (34.3)	35 (34.3)
Weight, kg, median (IQR)	9.5 (6.5–14.0)	5.2 (4.3–6.8)	8.3 (7.0–9.9)	11.5 (9.0–13.0)	20.0 (15.0–25.5)
Height, cm, median (IQR)	73.0 (61.0–96.0)	57.0 (52.0–63.4)	71.0 (63.0–75.0)	85.0 (73.0–91.5)	109.0 (99.3–123.5)
Medication information					
Linezolid dose, mg/kg/day, median (IQR)	30.0 (28.1–31.6)	30.0 (27.9–30.3)	30.0 (26.7–31.0)	30.0 (27.3–31.2)	30.0 (29.1–32.6)
Duration of treatment, day, median (IQR)	8 (4–15)	8 (4–14)	9 (4–15)	6 (4–15)	11 (5–17.5)
Clearance, L/h, median (IQR)	1.31 (1.29–1.32)	1.31 (1.30–1.32)	1.30 (1.07–1.31)	1.30 (1.04–1.32)	1.31 (1.30–1.32)
Exposure of linezolid					
AUC_24_, mg/L.h, median (IQR)	81.1 (60.6–108.7)	43.5 (34.1–53.3)	72.6 (64.9–76.9)	92.2 (85.5–100.0)	149.3 (122.1–189.4)

Abbreviations: AUC_24_, 24-h area under the concentration-time curve; IQR, interquartile range.

**TABLE 3:** Counts and percentages of the laboratory adverse events and the change over time before and after linezolid treatment according to the AUC_24_ quartile.

	Total	Quartile 1	Quartile 2	Quartile 3	Quartile 4
Laboratory adverse event, n (%)					
High TBil, umol/L					
Before medication	13/413 (3.1)	8/106 (7.5)	2/102 (2.0)	1/102 (1.0)	2/103 (1.9)
During treatment	25/374 (6.7)^a^	9/94 (9.6)	5/95 (5.3)	6/91 (6.6)	5/94 (5.3)
1–7 days after end of treatment	19/341 (5.6)^a^	10/86 (11.6)	2/86 (2.3)	3/80 (3.8)	4/89 (4.5)
8–15 days after end of treatment	11/292 (3.8)	7/67 (10.4)	1/74 (1.4)	1/72 (1.4)	2/79 (2.5)
High AST, U/L					
Before medication	26/413 (6.3)	10/106 (9.4)	6/102 (5.9)	8/102 (7.8)	2/103 (1.9)
During treatment	39/383 (10.2)^a^	14/97 (14.4)	6/95 (6.3)	11/96 (11.5)	8/95 (8.4)
1–7 days after end of treatment	26/352 (7.4)	9/86 (10.5)	3/90 (3.3)	9/85 (10.6)	5/91 (5.5)
8–15 days after end of treatment	23/314 (7.3)	5/73 (6.8)	5/78 (6.4)	8/81 (9.9)	5/82 (6.1)
High ALT, U/L					
Before medication	23/413 (5.6)	8/106 (7.5)	3/102 (2.9)	7/102 (6.9)	5/103 (4.9)
During treatment	48/380 (12.6)^a^	12/94 (12.8)	6/95 (6.3)	17/93 (18.3)	13/98 (13.3)
1–7 days after end of treatment	26/353 (7.4)	9/86 (10.5)	2/90 (2.2)	8/84 (9.5)	7/93 (7.5)
8–15 days after end of treatment	26/316 (8.2)	8/72 (11.1)	4/82 (4.9)	7/78 (9.0)	7/84 (8.3)
High Scr, mg/ml					
Before medication	11/413 (2.7)	6/106 (5.7)	1/102 (1.0)	3/102 (2.9)	1/103 (1.0)
During treatment	11/378 (2.9)	6/95 (6.3)	0/93 (0)	1/91 (1.1)	4/99 (4.0)
1–7 days after end of treatment	7/340 (2.1)	3/83 (3.6)	1/85 (1.2)	0/81 (0)	3/91 (3.3)
8–15 days after end of treatment	6/292 (2.1)	2/69 (2.9)	1/73 (1.4)	0/70 (0)	3/80 (3.8)
High BUN, mmol/L					
Before medication	4/413 (1.0)	2/106 (1.9)	0/102 (0)	1/102 (1.0)	1/103 (1.0)
During treatment	4/326 (1.2)	1/81 (1.2)	0/74 (0)	0/79 (0)	3/92 (3.3)
1–7 days after end of treatment	6/236 (2.5)	1/50 (2.0)	0/58 (0)	0/53 (0)	5/75 (6.7)
8–15 days after end of treatment	7/174 (4.0)	2/34 (5.9)	1/48 (2.1)	1/41 (2.4)	3/51 (5.9)
Thrombocytopenia, ×10^9^/L					
Before medication	23/413 (5.6)	3/106 (2.8)	4/102 (3.9)	7/102 (6.9)	9/103 (8.7)
During treatment	71/399 (17.8)^a^	17/104 (16.3)	15/98 (15.3)	18/97 (18.6)	21/100 (21.0)
1–7 days after end of treatment	102/399 (25.6)^a^	30/104 (28.8)	24/98 (24.5)	27/97 (27.8)	21/100 (21.0)
8–15 days after end of treatment	70/361 (19.4)^a^	5/86 (5.8)	10/94 (10.6)	21/90 (23.3)	34/91 (37.4)
Low hemoglobin, g/L					
Before medication	18/413 (4.4)	9/106 (8.5)	5/102 (4.9)	2/102 (2.0)	2/103 (1.9)
During treatment	61/401 (15.2)^a^	12/104 (11.5)	12/101 (11.9)	20/97 (20.6)	17/99 (17.2)
1–7 days after end of treatment	57/387 (14.7)^a^	12/99 (12.1)	7/96 (7.3)	14/95 (14.7)	24/97 (24.7)
8–15 days after end of treatment	54/350 (15.4)^a^	16/85 (18.8)	10/92 (10.9)	8/87 (9.2)	20/86 (23.3)
Low WBC, ×10^9^/L					
Before medication	24/413 (5.8)	2/106 (1.9)	4/102 (3.9)	7/102 (6.9)	11/103 (10.7)
During treatment	43/389 (11.1)^a^	7/101 (6.9)	9/98 (9.2)	13/91 (14.3)	14/99 (14.1)
1–7 days after end of treatment	49/383 (12.8)^a^	9/96 (9.4)	9/96 (9.4)	16/94 (17.0)	15/97 (15.5)
8–15 days after end of treatment	50/346 (14.5)^a^	6/84 (7.1)	11/90 (12.2)	13/85 (15.3)	20/87 (23.0)
Low neutrophil count, ×10^9^/L					
Before medication	8/413 (1.9)	0/106 (0)	2/102 (2.0)	2/102 (2.0)	4/103 (3.9)
During treatment	30/403 (7.4)^a^	2/103 (1.9)	5/99 (5.1)	9/98 (9.2)	14/103 (13.6)
1–7 days after end of treatment	22/385 (5.7)^a^	3/99 (3.0)	4/95 (4.2)	7/93 (7.5)	8/98 (8.2)
8–15 days after end of treatment	16/361 (4.4)^a^	2/84 (2.4)	2/91 (2.2)	5/92 (5.4)	7/94 (7.4)

Abbreviations: ALT, alanine amimotransferase; AST, aspartate aminotransferase; AUC_24_, 24 h area under the concentration-time curve; BUN, blood urea nitrogen; Scr, serum creatinine concentration; TBil, total bilirubin; WBC, white blood cell count.

a Significantly different from the before medication group by generalized estimation equation analysis (*p* < 0.05).

### Association Between LZD Exposure and Safety

The incidence of changes in the laboratory adverse events over time, before, and after medication is shown in Table 3. Adverse events associated with TBil, AST, ALT, PLT, hemoglobin, WBC, and neutrophil count increased during and after LZD treatment when compared with previous medication (*p* < 0.05). The association between LZD exposure and laboratory adverse events is shown in Table 4. The AUC_24_ quartile four group was associated with increased odds of low hemoglobin 1–7 days after LZD treatment compared with the quartile one group (adjusted OR: 4.768, 95% CI: 1.323-17.184, *p* = 0.017), and the AUC_24_ quartile three and quartile four groups were associated with increased odds of thrombocytopaenia 8–15 days after LZD treatment compared with the quartile one group (adjusted OR: 3.306, 95% CI: 1.126-9.709, *p* = 0.030 and adjusted OR: 3.770, 95% CI: 1.079-13.171, *p* = 0.038, respectively).

**TABLE 4:** Association between AUC_24_ and the laboratory adverse events during treatment, and 1–7 days, 8–15 days after the end of linezolid administration^a^.

Laboratory Outcomes	Continuous	Quartile 1	Quartile 2	Quartile 3	Quartile 4
During treatment					
High TBil, umol/L	1.012 (0.995–1.030)	Ref^b^	0.729 (0.205–2.588)	1.329 (0.369–4.795)	2.346 (0.327–16.836)
	*p* = 0.163		*p* = 0.625	*p* = 0.664	*p* = 0.396
High AST, U/L	0.998 (0.987–1.009)	Ref	0.401 (0.134–1.202)	0.553 (0.196–1.554)	0.221 (0.045–1.086)
	*p* = 0.694		*p* = 0.103	*p* = 0.261	*p* = 0.063
High ALT, U/L	1.000 (0.990–1.010)	Ref	0.577 (0.176–1.892)	1.635 (0.583–4.583)	0.839 (0.194–3.630)
	*p* = 0.823		*p* = 0.364	*p* = 0.350	*p* = 0.814
Thrombocytopenia, ×10^9^/L	0.995 (0.987–1.003)	Ref	0.727 (0.321–1.647)	0.679 (0.291–1.587)	0.429 (0.133–1.377)
	*p* = 0.201		*p* = 0.445	*p* = 0.372	*p* = 0.155
Low hemoglobin, g/L	1.001 (0.993–1.009)	Ref	1.378 (0.551–3.446)	2.227 (0.880–5.635)	1.249 (0.356–4.381)
	*p* = 0.889		*p* = 0.492	*p* = 0.091	*p* = 0.728
Low WBC, ×10^9^/L	0.996 (0.986–1.006)	Ref	1.354 (0.457–4.012)	2.114 (0.721–6.199)	2.324 (0.566–9.532)
	*p* = 0.388		*p* = 0.584	*p* = 0.173	*p* = 0.242
Low neutrophil count, ×10^9^/L	1.000 (0.992–1.009)	Ref	2.352 (0.439–12.604)	4.284 (0.864–21.241)	5.414 (0.881–33.277)
	*p* = 0.923		*p* = 0.318	*p* = 0.075	*p* = 0.068
1–7 days after end of treatment					
High TBil, umol/L	0.997 (0.975–1.019)	Ref	0.242 (0.040–1.482)	0.748 (0.152–3.693)	1.917 (0.191–19.212)
	*p* = 0.777		*p* = 0.125	*p* = 0.722	*p* = 0.580
Thrombocytopenia, ×10^9^/L	0.994 (0.986–1.001)	Ref	1.053 (0.535–2.071)	0.878 (0.425–1.815)	0.439 (0.154–1.251)
	*p* = 0.104		*p* = 0.882	*p* = 0.726	*p* = 0.123
Low hemoglobin, g/L	1.005 (0.997–1.014)	Ref	0.675 (0.201–2.261)	2.484 (0.863–7.152)	4.768 (1.323–17.184)
	*p* = 0.182		*p* = 0.524	*p* = 0.092	*p* = 0.017
Low WBC, ×10^9^/L	0.997 (0.988–1.006)	Ref	0.890 (0.319–2.486)	1.467 (0.547–3.933)	0.734 (0.193–2.793)
	*p* = 0.490		*p* = 0.824	*p* = 0.446	*p* = 0.650
Low neutrophil count, ×10^9^/L	1.001 (0.990–1.012)	Ref	1.294 (0.277–6.033)	2.398 (0.567–10.135)	2.664 (0.431–16.477)
	*p* = 0.870		*p* = 0.743	*p* = 0.234	*p* = 0.292
8–15 days after end of treatment					
Thrombocytopenia, ×10^9^/L	1.008 (1.001–1.016)	Ref	1.631 (0.522–5.095)	3.306 (1.126–9.709)	3.770 (1.079–13.171)
	*p* = 0.033		*p* = 0.400	*p* = 0.030	*p* = 0.038
Low hemoglobin, g/L	1.001 (0.993–1.009)	Ref	0.643 (0.244–1.694)	0.580 (0.204–1.649)	1.601 (0.457–5.608)
	*p* = 0.889		*p* = 0.371	*p* = 0.307	*p* = 0.462
Low WBC, ×10^9^/L	1.009 (1.001–1.017)	Ref	1.820 (0.630–5.255)	2.073 (0.705–6.093)	2.415 (0.638–9.146)
	*p* = 0.037		*p* = 0.269	*p* = 0.185	*p* = 0.194
Low neutrophil count, ×10^9^/L	1.003 (0.995–1.010)	Ref	0.813 (0.108–6.125)	2.373 (0.414–13.601)	4.970 (0.604–40.909)
	*p* = 0.509		*p* = 0.841	*p* = 0.322	*p* = 0.136

Values given are Odds ratios and 95% confidence intervals estimates.

a The binary logistic regression model was adjusted for variables including age (continuous, years), sex (male/female) and whether adverse events occurred before medication (yes/no).

b The Ref means taking quartile 1 as the reference category and comparing the data of quartile 2, quartile three and quartile four to those of quartile 1.

Abbreviations: ALT, alanine amimotransferase; AST, aspartate aminotransferase; AUC_24_, 24 h area under the concentration-time curve; TBil, total bilirubin; WBC, white blood cell count.

### Exposure Cut-Off Values for Laboratory Adverse Events

**FIGURE 2:**
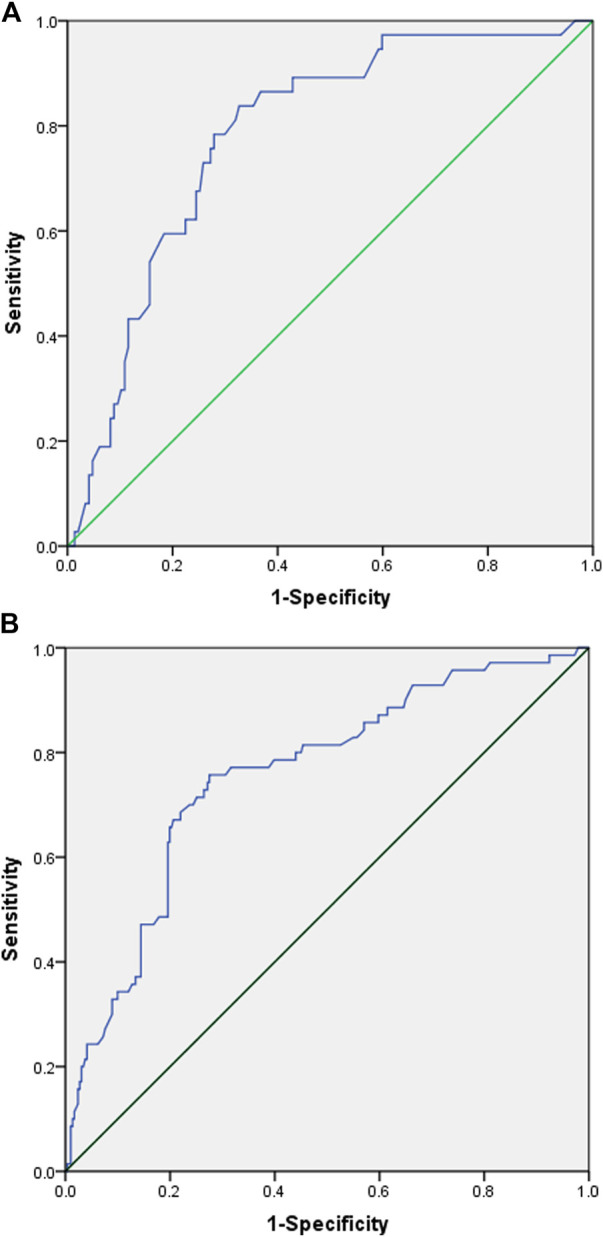
Receiver operating characteristic (ROC) curve of linezolid-induced adverse effect. **(A)** ROC curve of low hemoglobin 1–7 days after the end of linezolid administration. (Youden index = 0.511, cut-off values = 120.69, area under the ROC curve = 0.783, 95% confidence interval=(0.706-0.860), *p* < 0.001, sensitivity = 0.838, specificity = 0.673). **(B)** ROC curve of thrombocytopenia 8–15 days after the end of linezolid administration. (Youden index = 0.482, cut-off values = 92.88, area under the ROC curve = 0.756, 95% confidence interval=(0.693-0.820), *p* < 0.001, sensitivity = 0.757, specificity = 0.725).

An ROC analysis was subsequently performed to calculate the cut-off points of AUC_24_ for low hemoglobin and thrombocytopenia. The ROC curves and the associated results are shown in Figure 2. The cut-off with the largest Youden index of low hemoglobin 1–7 days after the end of LZD treatment was 120.69 mg/L h with a sensitivity of 83.8% and a specificity of 67.3%, and the cut-off with the largest Youden index of thrombocytopenia 8–15 days after the end of LZD treatment was 92.88 mg/L h with a sensitivity of 75.7% and a specificity of 72.5%.

### Efficacy Assessment

The most common site of infection was pulmonary [254 (61.5%)], followed by skin [99 (24.0%)], blood [90 (21.8%)], and endocarditis [83 (20.1%)](Supplementary Table S2). A total of 86.4% (357/413) of the included patients underwent bacterial culture before LZD treatment, and bacteria were found in 56.0% (200/357) of the patients. The species and MIC distributions of the bacterial strains isolated from patients before LZD treatment are presented in Table 5. Bacterial culture was performed in 68% (136/200) of the aforementioned patients by the end of the LZD therapy, and infections of 92.6% (126/136) of the patients were cleared, of whom 69.8% (88/126) of the patients had AUC_24_/MIC values greater than 80. Bacterial infections in 7.4% (10/136) of the patients were not cleared, of whom 90.0% (9/10) of the patients had AUC_24_/MIC values greater than 80.

**TABLE 5:** The species and MIC distribution of bacterial strains isolated from patients before linezolid treatment (n = 200).

Isolates	N (%)	MIC (Ug/mL)				
		≤0.5	≤0.064	≤1	≤2	4	
*Staphylococcus aureus*	78 (39.0)			12	65	1
*Staphylococcus* epidermidis	39 (19.5)			13	26	
*Streptococcus* *pneumoniae*	38 (19.0)	2		9	27	
*Human staphylococcus*	14 (7.0)			1	10	3
*Enterococcus faecium*	9 (4.5)				9	
*Streptococcus pallidus*	8 (4.0)	1	1		6	
*Enterococcus faecalis*	7 (3.5)	1		3	3	
*Staphylococcus haemolyticus*	3 (1.5)				3	
*Staphylococcus Coriolis*	3 (1.5)			1	2	
*Streptococcus pyogenes*	1 (0.5)			1		

MIC, minimal inhibitory concentration.

## Discussion

In this study, the patients who were intravenously or orally administered with LZD were all included, and the pharmacokinetic models of the corresponding route of administration were used to calculate the drug exposures. Studies have shown that there were no significant racial differences in the pharmacokinetic process of LZD in pediatrics (Jungbluth et al., 2003), so, we chose the pharmacokinetic model of oral administration, which was established based on a multiracial population, with race not considered a significant covariant (Garcia-Prats et al., 2019). Moreover, the pharmacokinetic model of intravenous administration of LZD was established based on Chinese pediatrics (Li et al., 2019).

We included patients with no “normal baseline values” in our study, as no “normal baseline values” does not mean it has reached the level of adverse events as defined in the study, and by comparing the incidence of associated adverse events before and after medication, we could see if there was a statistically significant increase in the incidence of related adverse events after medication. In fact, we did not find that patients with no “normal baseline values” were more prone to develop adverse events from our data. When analyzing the association between AUC_24_ and safety, laboratory parameters measured before LZD were considered as a potential confounding factor and were included as covariates in the binary logistic regression analyses, to avoid the influence of parameter differences between individuals before LZD medication on the statistical analysis, and to keep the validity of the results.

The hematological toxicity of LZD is widely known (Sasaki et al., 2011; Bayram et al., 2017), and in this study, thrombocytopenia and low hemoglobin were particularly significant. Pediatric patients with the treatment duration more than 28 days were more likely to have laboratory adverse events of low hematological indicators after using LZD. Therefore, hematological indicators should be carefully monitored during LZD treatment, especially for patients with long-term treatment (Dong et al., 2016). A previous study reported that one patient developed grade 4 neutropoenia 7 days after the end of LZD administration (Yasu et al., 2021), but the other influencing factors were unclear. In our study, low hemoglobin, thrombocytopenia, low WBC, and low neutrophil count occurred after the end of the LZD treatment in a significant proportion of the patients. The related mechanisms and other influencing factors deserve further study, but it seems a continuous period of monitoring after LZD withdrawal is also necessary.

Studies have reported that an adequate exposure to LZD was an AUC_24_ ranging between 160 and 300 mg/L h in adults (Pea et al., 2012; Cojutti et al., 2019), but the AUC_24_ cut-off value of LZD-associated thrombocytopenia was 280.7 mg/L h in adult patients (Pea et al., 2012) and 93.4 mg/L h for mitochondrial toxicity in infants and toddlers (Srivastava et al., 2016). In our study, we calculated the AUC_24_ cut-off values of 120.69 and 92.88 mg/L h for low hemoglobin and thrombocytopenia, respectively. It is suggested that for patients, especially pediatric patients, a trade-off is necessary between the level of drug exposure required for treatment and safety since an AUC_24_/MIC value greater than 80 is commonly recommended in clinics (Andes et al., 2002; Rayner et al., 2003; Pea et al., 2010; Li et al., 2019).

In accordance with the drug labels, the dosage of LZD was approximately 30.0 mg/kg/day for both intravenous and oral administrations in this study (U.S. Department of Health and Human Services, 2021), and we found that, although the infections in 92.6% (126/136) of the patients were cleared, 30.2% (38/126) of the patients had an AUC_24_/MIC value lower than 80. Since patient characteristics, peculiar pathophysiological conditions (e.g., cystic fibrosis, burn injuries, and sepsis), and combination medications could all affect the drug pharmacokinetic process and the apparent pharmacokinetic parameters (Di Paolo et al., 2010), and clearance of LZD in children younger than 12 years of age was greater than adults, with a correspondingly lower AUC_24_ (Jungbluth et al., 2003; Principi and Esposito, 2019), a previous study suggested that the LZD exposure target was an AUC_24_/MIC ratio of 62 with combination therapy (faropenem, LZD, and moxifloxacin) for disseminated and intrathoracic *tuberculosis* in infants and toddlers (Srivastava et al., 2016). Although concomitant medication was not a significant covariant in either of the two population pharmacokinetic models, when considering the efficacy, the exposure target of LZD in pediatrics might require further study, especially for pediatric patients with complications and concomitant medications.

This study had several limitations. First, the results were potentially only biased by the LZD that we analyzed being used at a limited centre. Second, our study only included patients younger than 13 years of age, limiting our ability to comprehensively assess LZD’s safety. Additionally, this study had a short follow-up period; therefore, large-scale, randomized clinical trials with longer follow-ups are still needed to further verify the safety and clinical efficacy of LZD.

## Conclusion

Hematological indicators should be carefully monitored during LZD treatment, especially thrombocytopenia and low hemoglobin, and a continuous period of monitoring after LZD withdrawal is also necessary. Since the AUC_24_ cut-off values for laboratory adverse events were relatively low, a trade-off is necessary between the level of drug exposure required for treatment and safety, and the exposure target (AUC_24_/MIC) in pediatrics should be further studied, especially for patients with complications and concomitant medications.

## Data Availability

The original contributions presented in the study are included in the article/Supplementary Material; further inquiries can be directed to the corresponding authors.

## Abbreviations

ALT, alanine aminotransferase; AST, aspartate aminotransferase; AUC24, 24 h area under the concentration-time curve; BUN, blood urea nitrogen; CIs, confidence intervals; HIS, hospital information system; IQR, interquartile range; LZD, linezolid; MIC, minimal inhibition concentration; NONMEM, nonlinear mixed-effects modeling program; ORs, odds ratios; PLT, platelet; ROC, receiver operating characteristic; TBil, total bilirubin; WBC, white blood cell count.
