# Supplementary material for: Relationship Between Linezolid Exposure and the Typical Clinical Laboratory Safety and Bacterial Clearance in Chinese Pediatric Patients
Source: Front Pharmacol. 2022 Aug 1;13:926711. doi: 10.3389/fphar.2022.926711 (PMC9377148; doi:10.3389/fphar.2022.926711)
Supplement: Supplementary file 1 [file Table1.docx]

| Supplementary Table 1: Characteristics of the population pharmacokinetic models referenced in this study | | |
| --- | --- | --- |
| Study | Pharmacokinetic modelling program | Formula |
| Garcia-Prats, AJ. et al ^[21]^ | NONMEM 7.3 | $K_{a}\left( h^{-1} \right)=0.77+\left( CL/V \right)$ |
|  |  | $CL/F\left( L/h \right)=4.73+\left( \mathrm{WT}\div70 \right)^{0.75}$ |
|  |  | $V/F\left( L \right)=54.8 \times\left( \mathrm{WT}\div70 \right)$ |
| Si-Chan Li, et al ^[22]a^ | NONMEM 7.41 | $CL=1.31\times\left( \frac{ln\mathrm{WT}}{2.40} \right)^{0.83}\times\left( \frac{ln\mathrm{eGFR}}{4.89} \right)^{0.60}$ |
|  |  | $V=4.24\times\left( \frac{ln\mathrm{WT}}{2.40} \right)^{0.86}$ |
|  |  | $\mathrm{eGFR}=\frac{k\times\mathrm{height}}{\mathrm{Scr}}$ |
| Abbreviations: CL: clearance; eGFR: estimated glomerular filtration rate; F: bioavailability; Ka: absorption rate constant; NONMEM: nonlinear mixed-effects modeling program; Scr: serum creatinine concentration. V: volume of distribution; WT: body weight in kilograms.  ^a^: eGFR was calculated according to the modified Schwartz formula; where k is 0.45 for infants <1 years, 0.55 for children <12 years and adolescent females, and 0.7 for adolescent males. | | |
