# Supplementary material for: Relationship Between Linezolid Exposure and the Typical Clinical Laboratory Safety and Bacterial Clearance in Chinese Pediatric Patients
Source: Front Pharmacol. 2022 Aug 1;13:926711. doi: 10.3389/fphar.2022.926711 (PMC9377148; doi:10.3389/fphar.2022.926711)
Supplement: Supplementary file 2 [file Table2.DOCX]

| Supplementary Table 2: The patients' site of infection based on Clinical diagnosis^a^ | |
| --- | --- |
| Site | n (%) |
| Pulmonary | 254 (61.5) |
| Skin | 99 (24.0) |
| Blood | 90 (21.8) |
| Endocarditis | 83 (20.1) |
| Intracranial | 62 (15.0) |
| Bone | 67 (16.2) |
| Sinusitis | 26 (6.3) |
| Upper respiratory tract | 15 (3.6) |
| Urinary tract | 9 (2.2) |
| Other^b^ | 25 (6.1) |
| ^a^:The number of infected sites in 135 patients was 1; The number of infected sites in 116 patients was 2; The number of infected sites in 92 patients was 3; The number of infected sites in 70 patients was more than 4.  ^b^:Including abdominal cavity, intestinal tract, laryngotracheobronchitis, bacteremia secondary to parotitis, empyema and catheter-associated infection. | |
